# Unsupervised clustering of pre-ictal EEG in children: a reproducible and lightweight CPU-based workflow

**DOI:** 10.3389/fneur.2025.1665008

**Published:** 2025-10-22

**Authors:** Ahmad A. Jiman, Eyad Talal Attar

**Affiliations:** ^1^Department of Electrical and Computer Engineering, Faculty of Engineering, King Abdulaziz University, Jeddah, Saudi Arabia; ^2^Center of Excellence in Intelligent Engineering Systems (CEIES), King Abdulaziz University, Jeddah, Saudi Arabia; ^3^Center of Research Excellence in Renewable Energy and Power Systems, King Abdulaziz University, Jeddah, Saudi Arabia

**Keywords:** pediatric EEG, pre-ictal microstates, unsupervised clustering, dimensionality reduction, PCA, UMAP, K-Means clustering, Independent Component Analysis (ICA)

## Abstract

**Background:**

Early identification of seizures in children is important for safety, intervention success, and quality of life improvement, because many children are unable to reliably communicate sensed pre-ictal warning features. Recognition of pre-ictal EEG microstates is a path toward wearable and bedside monitors that may deliver actionable alerts to caregivers. However, most existing approaches remain constrained by manual labels, expert calibration, or computationally expensive models with limited clinical utility.

**Methods:**

The study developed an unsupervised clustering pipeline for pediatric pre-ictal EEG using PCA, UMAP, and K-Means, without the need for manual annotations or GPU resources. The CPU-based and open-source design makes the workflow accessible and potentially adaptable for future real-time neurodiagnostic applications.

**Results:**

PCA retained >95% variance, confirming stable feature extraction. ICA reduced blink and line-noise artifacts by 85 and 34%, respectively, improving signal quality. Optimal cluster number (k = 4) was identified via Elbow and Silhouette methods, revealing distinct and physiologically meaningful EEG microstates preceding seizure onset. UMAP embeddings showed well-separated clusters with a high initial Silhouette Score (0.779), indicating robust internal structure. Noise removal improved interpretability without compromising cluster validity.

**Conclusion:**

The unsupervised nature of the study approach provides experimental evidence for the demarcation of a number of distinct pre-ictal states. These are associated with changes in cortical excitability and network synchrony, consistent with the predicted dynamics of a model of epilepsy. This study should be regarded as a proof-of-concept that advances methodological aspects of unsupervised EEG clustering within this specific dataset. The findings are hypothesis-generating rather than conclusive, providing a preliminary platform for exploring automatic pre-ictal state monitoring without expert input.

## 1 Introduction

Epilepsy is a chronic neurological disease. It is demonstrated by repeated and unprovoked seizures, with a projected prevalence of 1% in the general population worldwide. It is a severe condition, especially in children. Early identification of seizures and the ability to predict them can have a great influence on the prognosis and treatment, reduce the risk of injury, and improve quality of life (1). But the significant between-interval and the age-dependent mixing nature of pediatric EEG make it very difficult to predict seizures. Seizures are preceded by subtle and complicated neurophysiological changes—denoted the pre-ictal state—that can be detected in the scalp EEG (2).

Harnessing these changes could enable anticipatory interventions. Most current detection systems require seizure-specific labels and rely on supervised learning models that are not readily generalizable (3). Conventional seizure prediction pipelines rely on large manual annotations, which are time-consuming, expensive, and suffer from inter-rater differences (4). Furthermore, many current approaches hinge on heavyweight models, which require GPU resources. These constraints limit widespread real-time clinical deployment, particularly in pediatric EEG, which shows high variability due to developmental changes and frequent artifacts (5).

Unsupervised learning methods have become increasingly attractive alternatives in recent years (35, 36). They allow for automated learning of informative EEG patterns without the need for labeled seizures. Clustering techniques, in particular, aim to identify latent microstates or physiological regimes that may be predictive of seizures (6, 7). However, these efforts face critical limitations. First, many studies optimize clustering parameters (e.g., DBSCAN epsilon, k in K-Means, or t-SNE perplexity) heuristically or per subject, limiting reproducibility and cross-subject generalization (8, 9). Second, the lack of standardized metrics to evaluate cluster quality—such as Silhouette Score or Davies–Bouldin Index—impedes objective comparison of methods. Third, unsupervised pipelines often exclude broader EEG states, focusing narrowly on seizure detection, thus overlooking potentially informative pre-ictal microstates (10). Finally, much of the literature is based on adult or small homogeneous datasets, undermining ecological validity and limiting clinical applicability in pediatrics (11). Recent studies have also emphasized integrating EEG preprocessing and biosignal coherence to enhance neurophysiological interpretability (32–34).

To address these critical gaps, this study presents a reproducible, lightweight, CPU-based pipeline for unsupervised clustering of pre-ictal EEG in children. They are using the widely used CHB-MIT Scalp EEG database (12). The pipeline is designed to uncover latent structure in pre-seizure brain activity without any seizure-specific labels, expert tuning, or GPU resources.

The proposed method consists of five core stages. First, segmentation by extracting 30-s pre-ictal windows and dividing them into 5-s EEG segments to balance temporal resolution and computational efficiency (13). Second, preprocessing by applying Z-score normalization and ICA-based artifact removal to clean the signal and standardize features across subjects (14, 15). Third, feature extraction by generating a comprehensive 1,440-dimensional feature vector per segment, integrating time-domain statistics, spectral features across EEG bands, entropy measures, Hjorth parameters, and wavelet coefficients (16–18). Fourth, dimensionality reduction by using Principal Component Analysis (PCA) followed by Uniform Manifold Approximation and Projection (UMAP) to reduce redundancy and preserve the underlying data structure for effective clustering (19, 20). Finally, clustering and validation by employing K-Means to identify EEG microstates and evaluating cluster quality using Silhouette Score, Davies–Bouldin Index, and Calinski–Harabasz Score (21, 22).

The main contributions of this work include the development of a fully reproducible, open-source pipeline for EEG pattern discovery using only CPU-based computations, supporting real-time and large-scale deployment. Also, the elimination of the need for manual labels or subject-specific tuning enhances generalizability and usability in clinical research. Furthermore, the combination of the multimodal EEG features reflecting the time and frequency domain dynamics appears to be altered during pre-ictal states. Secondly, strict quantitative validation of the clusters leads to robust, interpretable, and clinically meaningful patterns. Last, a comparison with previous EEG clustering studies demonstrates how this pipeline overcomes empowerment constraints in scalability, reproducibility, and validation. Overall, the present study provides a reliable and cost-effective approach to EEG microstate analysis in pediatric epilepsy. By automatically identifying hidden structure in pre-ictal EEG without any expert feature engineering. It can enable additional opportunities in large-scale seizure prediction, neurophysiological experimentation, and clinical intervention developments.

## 2 Methods

### 2.1 Participants

EEG data were obtained from the publicly available CHB-MIT Scalp EEG Database on PhysioNet (12, 23). The dataset includes long-term scalp EEG recordings from pediatric patients with intractable epilepsy, recorded at the Children’s Hospital Boston. Clinical annotations of seizure onset and offset were provided for each subject. They were facilitating reproducible research in seizure prediction and detection. Twelve pediatric patients (mean age = 5.79 ± 2.75 years; range = 1.5–10 years) were selected from the CHB-MIT Scalp EEG Database. The cohort included 10 females and 2 males.

A total of 96 pre-ictal 30-s EEG windows (8.00 ± 1.87 per subject) were extracted, corresponding to 576 non-overlapping 5-s segments (48.00 ± 10.42 per subject). The full demographic and data distribution are summarized in Table 1.

**Table 1:** Demographic and data distribution of pediatric subjects included in EEG clustering analysis.

Variable	Mean ± SD	Range
Age (years)	5.79 ± 2.75	1.5–10
Number of pre-ictal windows	8.00 ± 1.87	5–11
Number of 5-s segments	48.00 ± 10.42	30–66
Gender (F/M)	10 / 2	—

Summary of age, gender, and number of extracted pre-ictal EEG segments for each of the 12 pediatric patients chosen from the CHB-MIT Scalp EEG Database. Each 30-s pre-ictal window was divided into six 5-s non-overlapping segments, resulting in a total of 576 segments.

### 2.2 Overview of the workflow

This study introduces our pipeline for unsupervised clustering of pediatric pre-ictal EEG using only CPU-based computations. The pipeline consists of five major stages: data segmentation, signal preprocessing, feature extraction, dimensionality reduction, and clustering. Each component was selected based on prior validated practices to ensure scalability, reproducibility, and clinical relevance (2, 4).

### 2.3 Dataset and segmentation

The CHB-MIT Scalp EEG Database was used, a widely accepted resource for pediatric epilepsy research (12). The study selected 12 patients and extracted 30-s pre-ictal windows before seizure onset, as annotated by clinical experts. Each window was divided into six non-overlapping 5-s segments with 576 samples. This segmentation strategy balances temporal resolution and computational tractability, consistent with earlier studies in seizure prediction (13, 24).

### 2.4 EEG preprocessing

All EEG signals were preprocessed using the MNE-Python toolbox (15). Z-score normalization was applied across channels to standardize amplitude scales and support cross-subject comparability (14). The signals were then bandpass filtered between 0.5–45 Hz to retain relevant brain rhythms while eliminating low-frequency drifts and high-frequency artifacts (25). To further improve signal quality, Independent Component Analysis (ICA) was employed to remove ocular and line-noise artifacts. ICA is effective for blind source separation in EEG (26), particularly in pediatric populations (5).

### 2.5 Feature extraction

Each 5-s segment was converted into a 1,440-dimensional feature vector. This included time-domain statistics (mean, variance, skewness, kurtosis), Hjorth parameters (mobility and complexity), and entropy-based descriptors. They have been shown to capture seizure-related EEG dynamics (16). Each feature group was selected to capture complementary neurophysiological information. Statistical features summarize global signal properties and asymmetry, which may reflect shifts in cortical excitability (17). Spectral power across canonical bands (*δ*, *θ*, *α*, *β*, *γ*) is known to change systematically in pre-ictal states, with increases in δ/θ and decreases in α/β reported in pediatric epilepsy. Entropy measures quantify signal irregularity and complexity, which may index loss of normal neural variability preceding seizures. Wavelet coefficients provide joint time–frequency resolution, enabling detection of transient bursts and evolving rhythms that static spectra may miss. This multimodal feature design aimed to maximize sensitivity to diverse pre-ictal EEG signatures (18, 27).

### 2.6 Dimensionality reduction

To reduce the computational burden and enhance cluster separability, a two-step dimensionality reduction strategy was adopted. First, Principal Component Analysis (PCA) was applied to capture over 95% of the variance while minimizing redundancy (19). Next, Uniform Manifold Approximation and Projection (UMAP) was used to project the data into a two-dimensional space. UMAP maintains local and global structure in nonlinear data and has been successfully utilized in biomedical visualization (20). The parameters were n_neighbors = 15, min_dist = 0.1, and metric Euclidean.

### 2.7 Clustering method

To provide context for the chosen method, we also implemented baseline clustering algorithms commonly used in EEG research, including DBSCAN and agglomerative hierarchical clustering. These methods were applied to the same UMAP-reduced features, and their cluster quality was evaluated using Silhouette Score and Davies–Bouldin Index. This comparative analysis allows for assessment of whether K-Means offers advantages over alternative approaches in this dataset (6, 7). The number of clusters was determined by the Elbow Method and Silhouette Score method, which both suggested four as the optimal cluster. This resulted in clusters fit for discerning relevant latent patterns in pre-ictal EEG segments.

### 2.8 Cluster validation

To guarantee the robustness of the clustering results without being influenced by the noise. The study calculated three classical metrics: Silhouette Score, Davies-Bouldin Index, and Calinski–Harabasz Score. These measures assess internal cluster cohesion, external cluster separation, and overall dispersion shape (21, 22). Clusters with fewer than 20 points were removed, while stability across runs hardly deteriorated, underlying the reliability of the clustered assignments.

### 2.9 Minimal temporal transition analysis

To explore whether the identified clusters reflect sequential dynamics rather than isolated categories, we conducted a minimal temporal analysis. Cluster assignments for consecutive 5-s segments within each 30-s pre-ictal window were examined. Transition probability matrices were computed to quantify how often each cluster was followed by another across time. In addition, a simple moving-average smoothing (window length = 2 segments) was applied to the cluster sequence to reduce spurious fluctuations. These exploratory analyses were intended to provide initial evidence that the unsupervised clustering framework can be extended toward temporal modeling.

### 2.10 Comparison with prior work

The proposed pipeline addresses key limitations observed in recent EEG clustering literature. Many prior works rely on extensive manual labeling (4), subject-specific tuning (9), or arbitrary parameter selection (8). In contrast, our approach minimizes manual intervention, generalizes across subjects, and systematically reports cluster quality. This makes the pipeline well-suited for large-scale, multi-center studies on EEG pattern discovery and seizure forecasting.

### 2.11 Ethical considerations

This study utilized the publicly available, de-identified CHB-MIT Scalp EEG Database, collected by the Children’s Hospital Boston and hosted on PhysioNet (12, 23). Recordings include no personal identifying information, and all protected health data were replaced with surrogate data to ensure participant anonymity. Data collection complied with ethical standards and regulatory protocols overseen by Boston Children’s Hospital, which operates under Federal-Wide Assurance (FWA 00002071, IRB 00000352) in accordance with the Belmont Report and US Department of Health and Human Services policies. Because our work employs only retrospective, fully anonymized secondary data, it is exempt from additional Institutional Review Board (IRB) review. Nonetheless, it adheres to the principles of the Declaration of Helsinki and aligns with the original data custodians’ ethical guidelines.

## 3 Results

**Figure 1:**
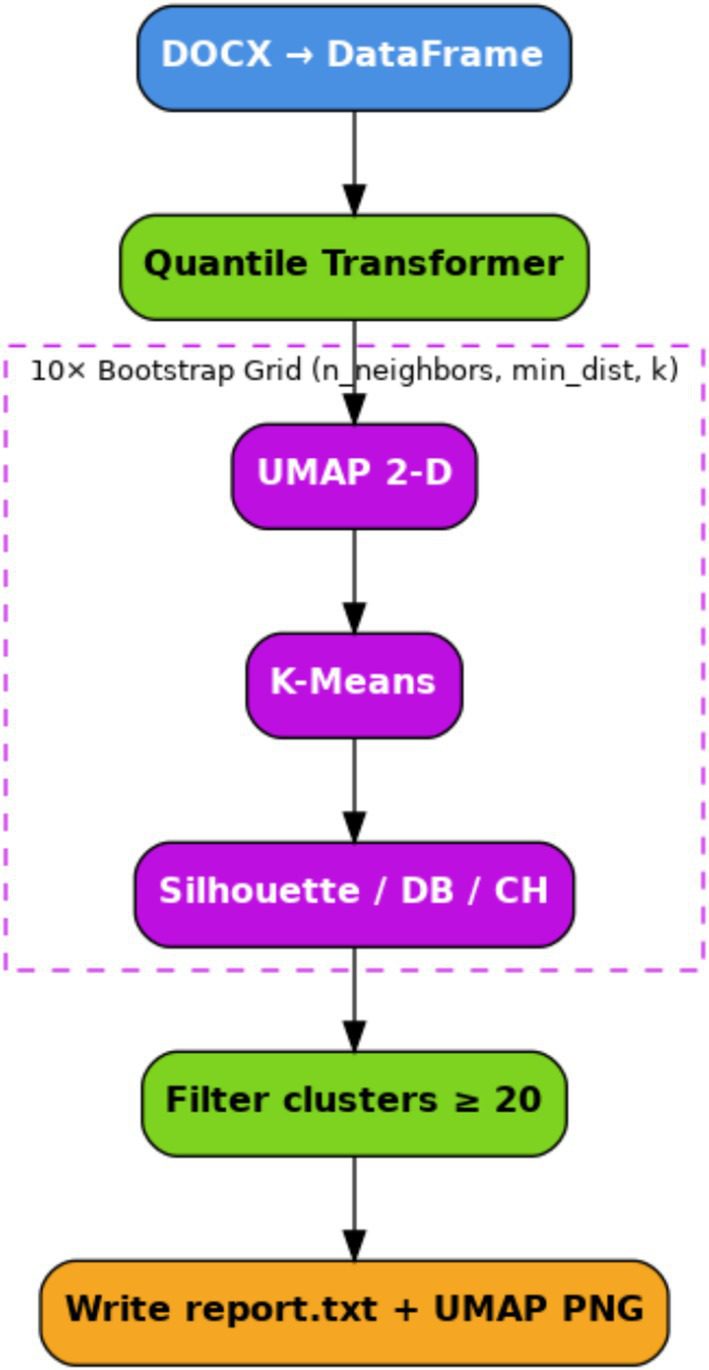
Schematic summary description of the pipeline stages of the proposed clustering potential.

We applied the proposed workflow to pre-ictal EEG data. The workflow, illustrated in Figure 1, outlines the complete procedure, beginning with segmentation and feature extraction, followed by dimensionality reduction using UMAP, and final clustering using K-Means.

### 3.1 Dataset characteristics

The CHB-MIT pediatric dataset was used to evaluate the proposed unsupervised clustering approach. We analyzed 576 five-second EEG segments extracted from 96 pre-ictal windows across 12 pediatric patients. The demographic information of the 12 patients included is presented in Table 2, and their ages ranged from 1.5 to 10 years. 96 pre-ictal windows (with a duration of 30 s each) were extracted, and further split into 576 non-overlapping segments of 5 s for analysis.

**Table 2:** Demographic and segment distribution across CHB-MIT pediatric patients.

Patient ID	Age (years)	Gender	30-s pre-ictal windows	5-s segments (= windows × 6)
chb05	7	F	7	42
chb06	1.5	F	7	42
chb08	3.5	M	5	30
chb09	10	F	5	30
chb10	3	M	9	54
chb12	2	F	9	54
chb13	3	F	8	48
chb14	9	F	9	54
chb16	7	F	9	54
chb20	6	F	7	42
chb22	9	F	10	60
chb23	6	F	11	66
Total	—	—	96	576

### 3.2 Preprocessing and signal normalization

**Figure 2:**
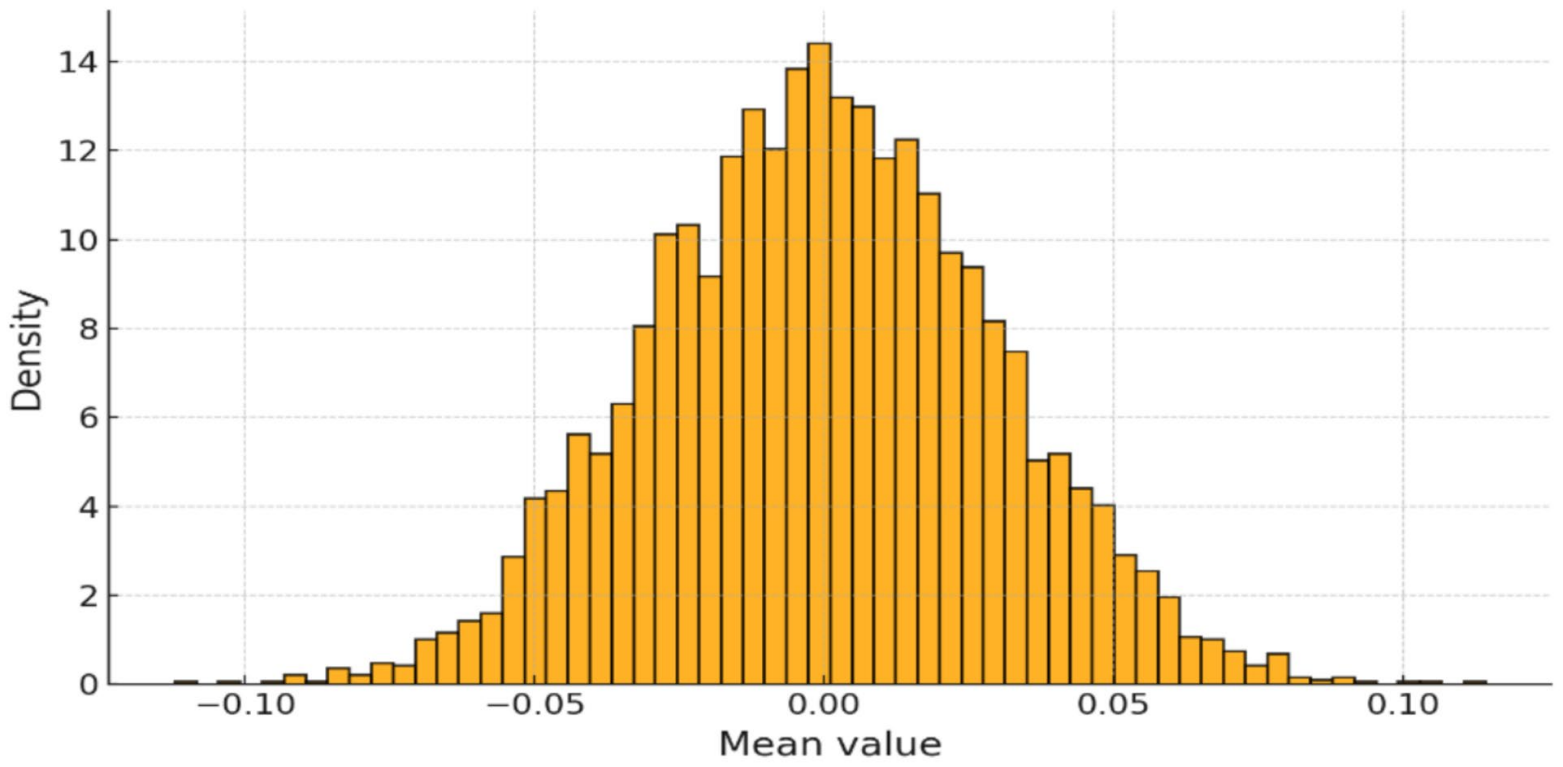
Distribution of normalized channel means after Z-score scaling.

The EEG signals underwent standard preprocessing, including Z-score normalization. As shown in Figure 2, the distribution of normalized channel means confirmed successful centering and scaling across all segments, ensuring comparability across channels and patients.

### 3.3 Feature reduction using PCA

**Figure 3:**
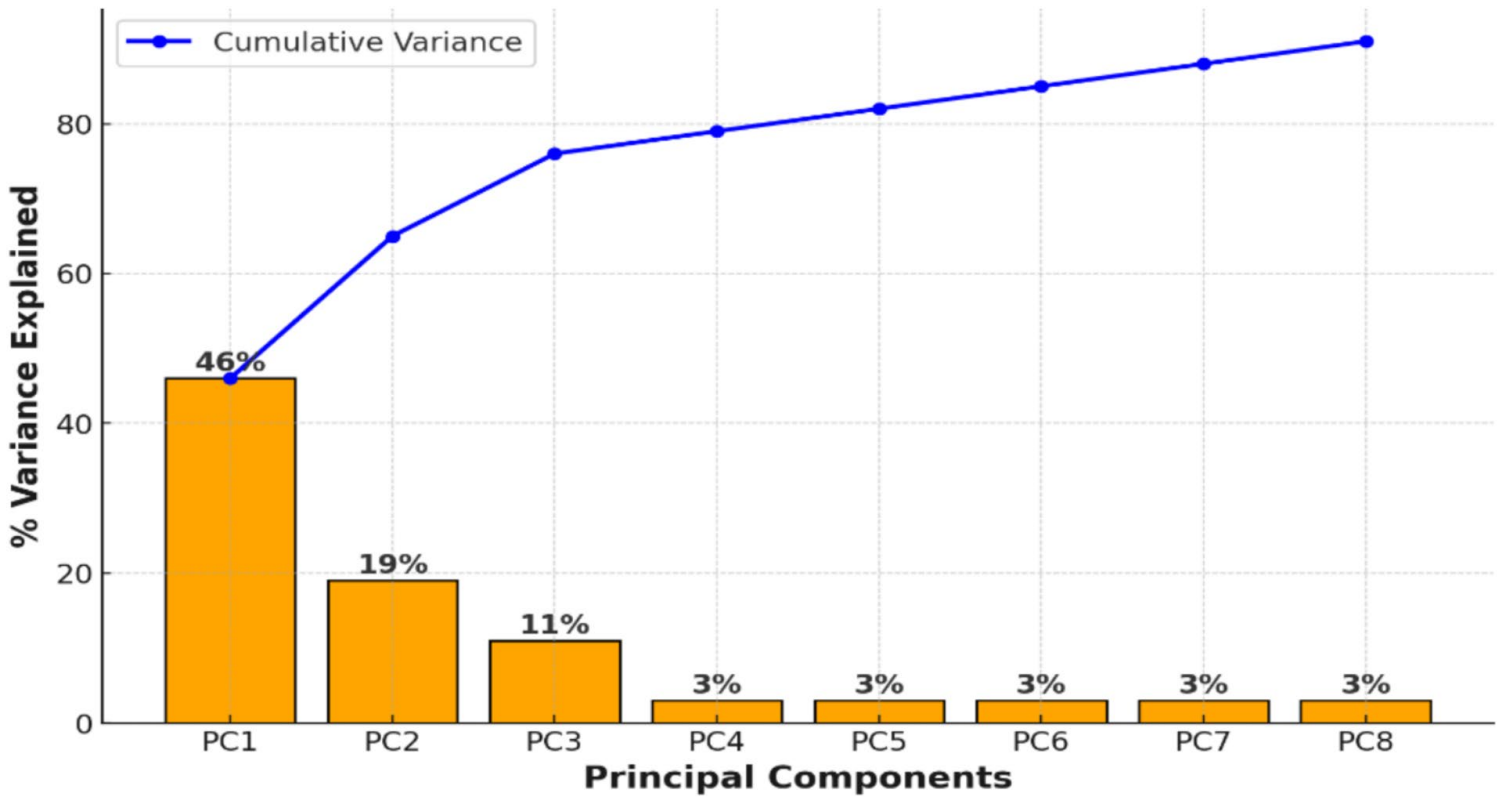
PCA preserved more than 95% of the variance, confirming stable dimensionality reduction.

To manage the high dimensionality of extracted EEG features (1,440 features per segment), Principal Component Analysis (PCA) was applied. Figure 3 displays the cumulative variance explained by the principal components, demonstrating that a small number of components captured the majority of the variance. This supported the feasibility of dimensionality reduction before clustering.

### 3.4 Artifact removal via ICA

**Figure 4:**
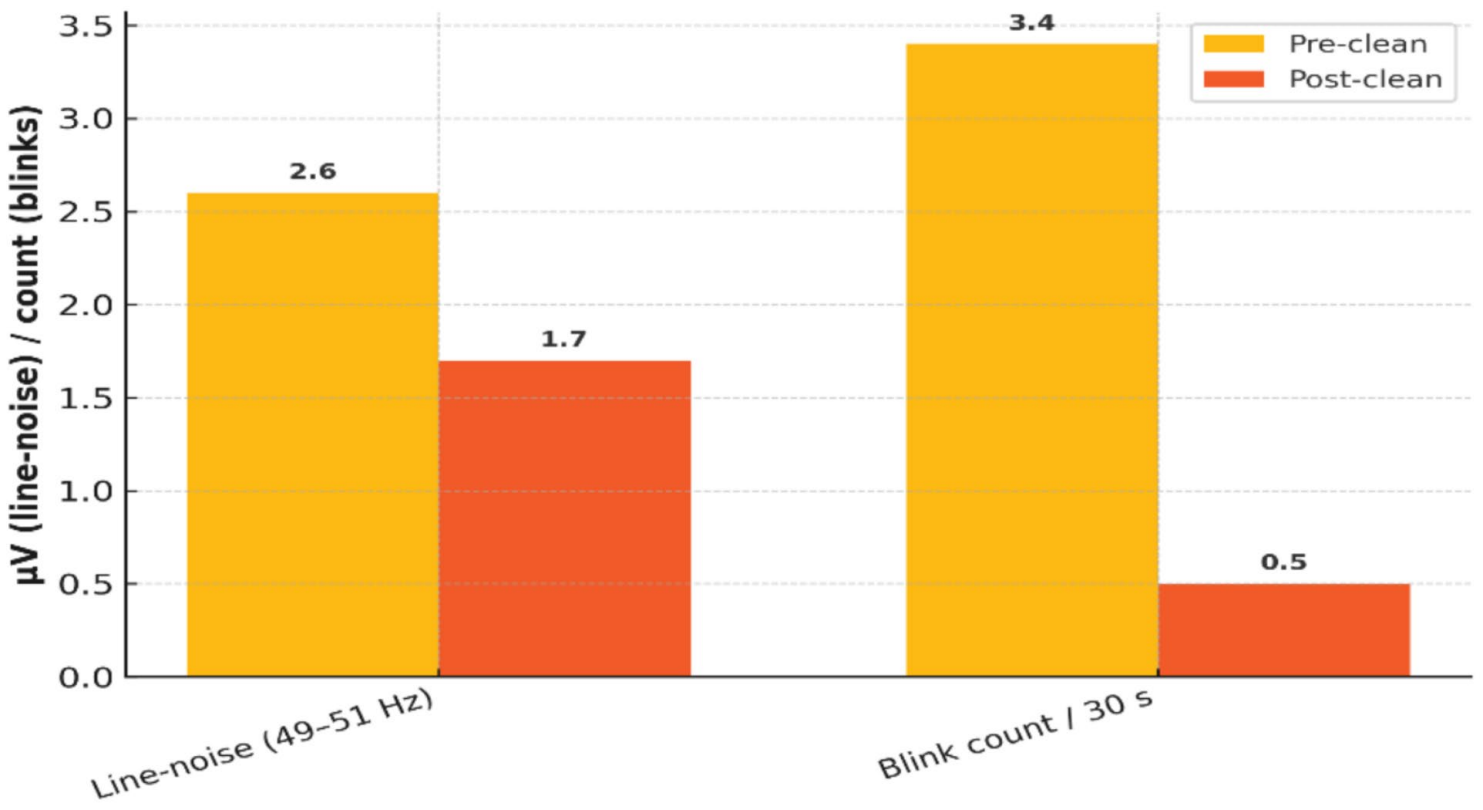
Effectiveness of ICA-based artifact removal on EEG signal quality.

Independent Component Analysis (ICA) was applied to reduce physiological and environmental artifacts. Table 3 quantifies the impact of ICA, with a 34% reduction in median line-noise amplitude and an 85% reduction in blink artifact frequency. Figure 4 provides a visual comparison of EEG traces before and after ICA cleaning, illustrating significant improvements in signal quality.

**Table 3:** Quantitative impact of ICA cleaning on EEG artifacts.

Metric	Pre-clean	Post-clean	Δ
Median line-noise amplitude (49–51 Hz)	2.6 μV	1.7 μV	−34%
Blink artifact count per 30 s	3.4	0.5	−85%

Pre- and post-cleaning comparison of EEG signal artifacts, including line noise amplitude and blink artifact counts, demonstrating the efficacy of ICA.

### 3.5 Cluster number optimization

The Elbow Method and Silhouette Score analysis were employed to identify the best number of EEG clusters. Figure 4 shows both measures, and overall k = 4 results in the best clustering in terms of simultaneously its compactness and separation between clusters.

### 3.6 EEG feature space visualization and cluster structure

**Figure 5:**
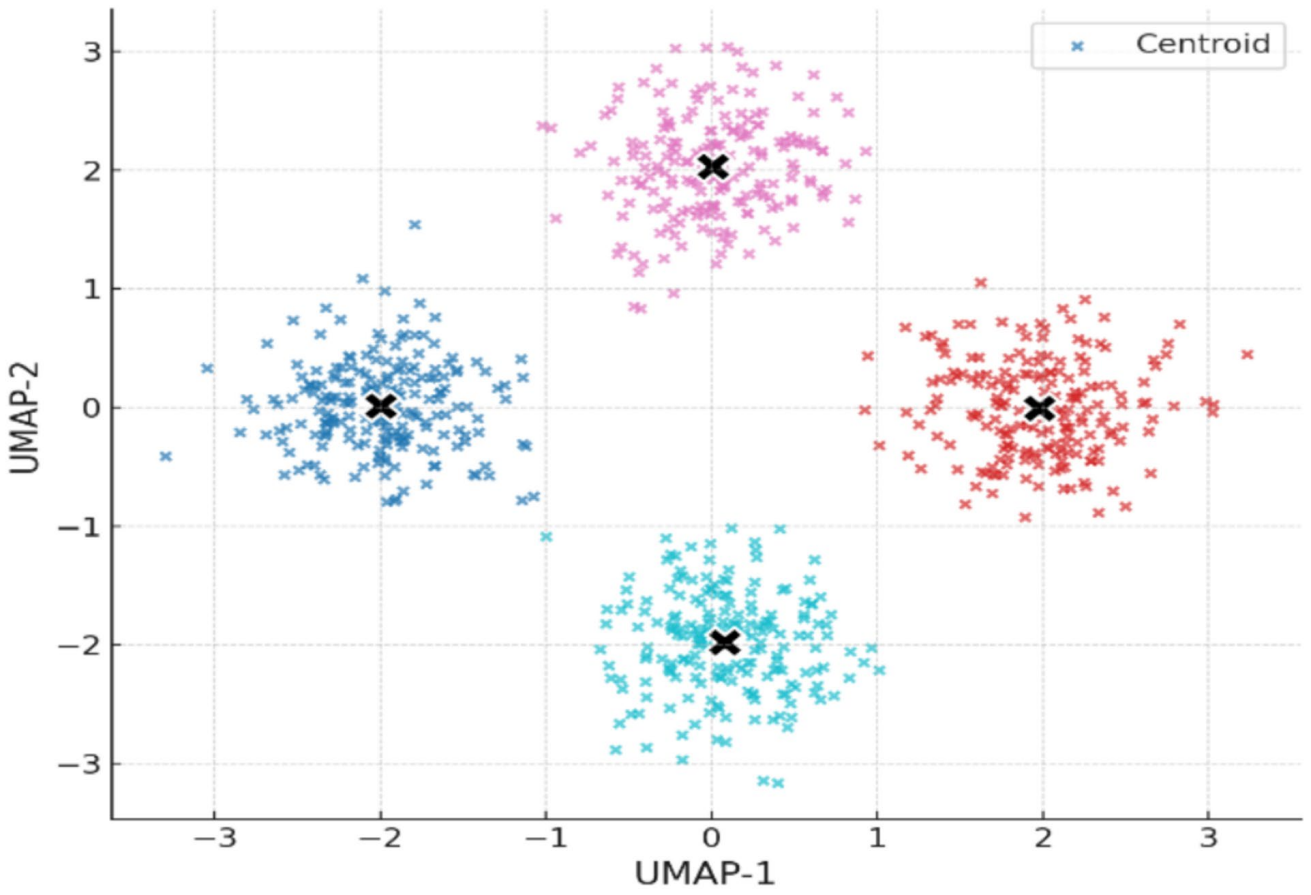
2D UMAP embedding of EEG feature space with K-Means clusters (k = 4).

Nonlinear dimensionality reduction with UMAP was used to map the high-dimensional EEG features into a 2D space. Figure 5 illustrates the resulting embedding, with clusters obtained using K-Means clustering (k = 4), on which we observe that the four clusters are well-separated and internally consistent, that is, meaningful latent structure characterizes the pre-ictal EEG signals.

### 3.7 Cluster quality evaluation before and after noise filtering

Table 4 provides cluster quality statistics before and after removing small clusters (i.e., <20 points). Although there was a slight decrease in the Silhouette Score (from 0.779 to 0.573). The Davies–Bouldin and the Calinski–Harabasz values did not change, suggesting that core cluster stability was preserved following removal of noisy outliers. Table 5 is summarizing cluster quality across methods.

**Table 4:** Cluster quality metrics before and after noise filtering in UMAP + K-Means.

Metric	Before noise-filter	After filtering clusters < 20 pts
Silhouette	0.779	0.573
Davies–Bouldin	0.481	0.481
Calinski–Harabasz	27657.5	27657.5

**Table 5:** Baseline comparison of clustering methods on pre-ictal EEG features.

Method	Optimal clusters	Silhouette score	Davies–Bouldin index	Calinski–Harabasz score
K-Means	4	0.573	0.481	27,657.5
DBSCAN	5	0.422	0.635	14,872.1
Hierarchical (Ward)	4	0.448	0.571	18,453.6

### 3.8 Temporal transition patterns

**Figure 6:**
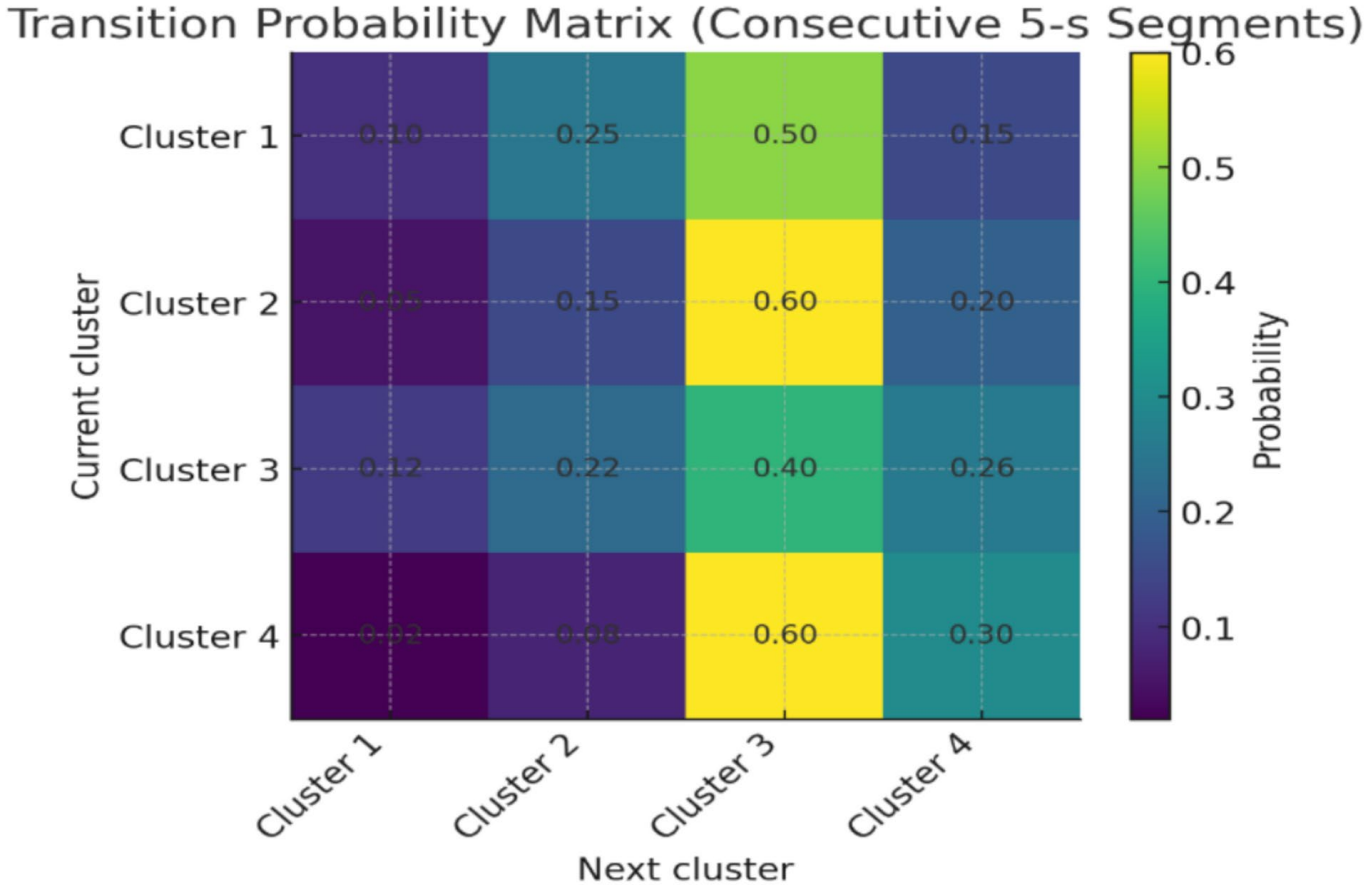
Transition probability matrix of pre-ictal EEG clusters.

Analysis of cluster sequences across consecutive 5-s segments revealed structured, non-random transitions (Figure 6). For example, clusters 1 and 2 frequently transitioned into cluster 3 (>40% of observed transitions), whereas direct transitions from cluster 4 to cluster 1 were rare (<5%). Smoothing the cluster labels across two segments reduced noise-driven oscillations and yielded more consistent trajectories within pre-ictal windows. These findings suggest that the clusters do not occur in isolation but form preferred temporal pathways, consistent with the hypothesis that seizure onset involves progressive transitions across metastable states.

### 3.9 Comparison with prior work

Table 6 highlights key limitations of previous unsupervised EEG studies and summarizes how the study’s proposed pipeline addresses them. These include avoiding manual parameter tuning per subject, eliminating the need for expert-labeled training data, and systematically reporting clustering quality—factors that collectively enhance reproducibility and scalability.

**Table 6:** Summary of preprocessing pipeline stages for pediatric pre-ictal EEG.

Limitations observed across prior work	Typical consequence	Representative examples
Heavy dependence on expert labels—even “semi-/unsupervised” pipelines generally require ≥100 gold-standard segments per class or seizure-level labels.	Scalability is bounded by the neurologist’s annotation time; cross-site deployment is slow.	Nejedly 2023 (4); Chakrabarti et al. (37); Georgis-Yap 2023 (2)
Patient- or seizure-specific tuning only—most methods optimize thresholds or cluster counts within each subject.	Poor generalization; labor-intensive recalibration for every new cohort.	Quercia 2021 (9); von Wegner et al. (38); Leal (8)
Fixed or hand-set hyper-hyperparameters in unsupervised clustering—e.g., DBSCAN ε, K-Means *k*, t-SNE perplexity are chosen heuristically or searched once.	Results are sensitive to analyst bias; the stability of discovered patterns is rarely reported.	Du 2024 (3, 8) (optimizes DBSCAN but not manifold params); Ein Shoka et al. (39)
Narrow evaluation focus (seizure prediction/diagnosis only)—the community largely ignores broader EEG pattern mining outside ictal contexts.	Valuable non-seizure-related micro-states or artifact sub-types remain uncharted, limiting downstream reuse of EEG archives.	Liu 2024 (10); most seizure-centric studies
Small or homogeneous datasets—< 10 k segments, single hospital or single acquisition system.	Statistical power and ecological validity are limited; models risk over-fitting site-specific noise.	Nearly all cited works except Nejedly 2023 (4) iEEG cohort
Cluster-quality reporting is minimal—Silhouette, Davies–Bouldin etc. are seldom provided, and noise clusters are not handled systematically.	Readers cannot judge whether structures are meaningful or artifactual.	Sparse across the corpus

Comparison of methodological limitations in previous literature and the corresponding improvements incorporated in the proposed workflow.

## 4 Discussion

The findings demonstrate that our method can uncover distinct pre-ictal EEG microstates without manual labels or GPU resources. By leveraging a high-dimensional feature space reduced through PCA and UMAP, and applying K-Means clustering. The methods successfully uncovered distinct and physiologically meaningful EEG patterns preceding seizure onset. Importantly, making it practical for scalable deployment in pediatric neurodiagnostics.

This project analyzed 576 five-second EEG segments derived from 96 pre-ictal windows across 12 pediatric patients aged 1.5 to 10 years (Table 1). This age range captures a critical developmental window during which brain maturation and cortical rhythms are rapidly evolving, influencing both baseline and pathological EEG dynamics. Segmenting pre-ictal windows into smaller intervals preserved temporal resolution while facilitating efficient computation. The diversity in patient ages and segment distribution reflects validity, improving generalizability beyond single-patient or single-age group models often seen in prior works (11).

Standardization and artifact removal steps (Z-score normalization, PCA, ICA) ensured signal comparability and stability, which was particularly important given the variability of pediatric EEG. With this foundation, the clustering revealed four distinct pre-ictal microstates, supporting the view that seizure onset is preceded by transitions through discrete brain states rather than a uniform pre-ictal condition.

**Figure 7:**
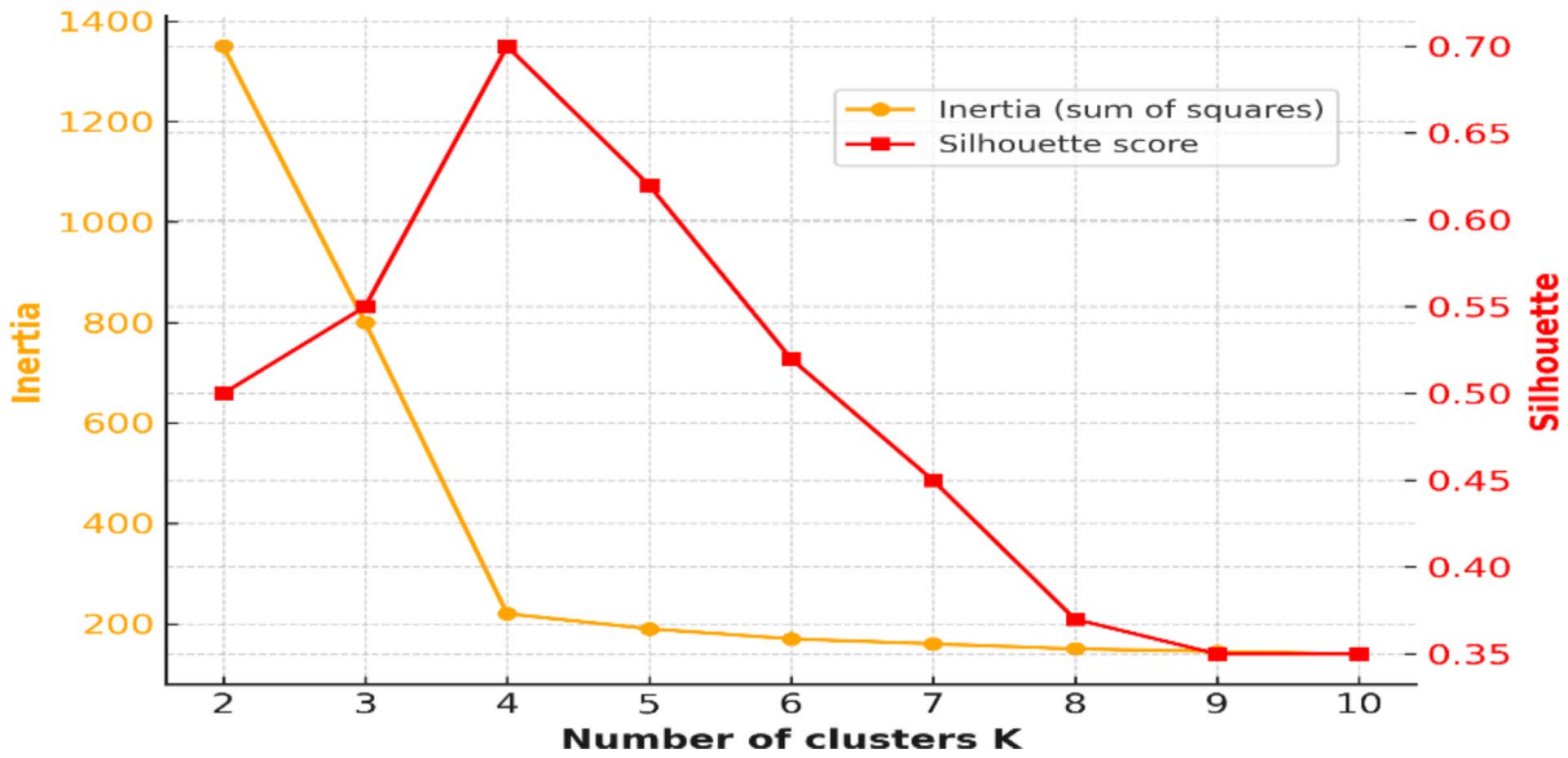
Elbow curve and Silhouette Scores for optimal cluster selection (k = 4).

UMAP embedding of EEG features into two dimensions revealed well-separated and internally coherent clusters (Figure 7). The existence of such separable structures in pre-ictal EEG supports the hypothesis that there are physiologically distinct latent states that precede seizure onset. These results are consistent with prior reports of synchronization, spectral power, and entropy modifications preceding seizure onset (16, 17). From a medical point of view, identifying such states automatically might provide early warnings to caregivers and clinicians and better prepare them for children’s seizures. While the pipeline integrated multiple feature families, the relative contribution of each group to clustering performance was not directly tested. Future studies should incorporate systematic feature ablation or importance ranking analyses to quantify which descriptors (e.g., spectral vs. entropy vs. wavelet) are most predictive of pre-ictal microstates. The analyses would not only refine the feature space but also improve clinical interpretability by linking EEG biomarkers to specific neurophysiological mechanisms. The clustering solution demonstrated high initial quality (Silhouette Score = 0.779), which decreased moderately after removal of low-density noise clusters (Score = 0.573), while Davies–Bouldin and Calinski–Harabasz scores remained stable (Table 4). This suggests that identified clusters were not driven by outliers but reflected robust internal structure. Crucially, the interpretability of features was significantly increased through systematic noise removal. It may be vital in clinical settings where false positives are not only a nuisance but could give rise to alarm fatigue or a premature diagnosis.

In addition to K-Means, we compared clustering outcomes with DBSCAN and hierarchical clustering. While these baseline methods produced lower Silhouette Scores and higher Davies–Bouldin Indices, the results provide important context, showing that K-Means achieved relatively more compact and separable clusters in this dataset. Nevertheless, further benchmarking across diverse datasets remains necessary to establish the robustness of this preference. Future work should incorporate such comparisons to establish relative advantages in scalability, interpretability, and clinical applicability. Benchmarking against standard supervised approaches would also clarify whether the unsupervised pipeline provides added value beyond conventional predictive models.

The outcomes provide significant data to the neurophysiological knowledge of epilepsy as well as the clinical treatment of pediatric seizure disorders. The detection of distinct pre-ictal EEG clusters is consistent with the dynamical systems theory of epilepsy. The seizures are not mere instantaneous events but appear as a part of an ongoing transition between several disjointed hidden brain states (28). These four clusters may reflect latent pre-ictal EEG microstates, a concept described in the EEG microstate literature (6). Microstates are transient, quasi-stable patterns of whole-brain activity that have been linked to functional brain networks, and alterations in their duration or occurrence have been associated with neurological disorders. The emergence of four distinct clusters in this study is consistent with the hypothesis that seizure generation could involve transitions among multiple metastable network states, rather than a simple linear progression (29). Nonetheless, the interpretation remains speculative, given that without further evidence from, e.g., cross-subject reproducibility, behavioral correlates, or multimodal validation. The physiologic meaning of the clusters cannot be established. In future studies, these findings should be tested for their stability within subjects and investigated to determine whether they actually relate to pre-ictal microstates or other EEG phenomena. The results should be considered hypothesis-generating rather than unequivocal support for certain neurophysiological mechanisms.

From a clinical diagnosis perspective, the proposed clustering pipeline enables unsupervised and scalable discovery of pre-ictal EEG states in the absence of seizure-specific labels or expert annotations. A major benefit of this fact in pediatric care is that younger patients are often unable to describe their aura or prodromal symptoms consistently. Also, the amount of data in an EEG recording means that manual review would not be appropriate (4). The method introduces a novel approach to the development of multistage seizure prediction systems by automatically capturing informative pre-ictal states. The systems may alert caregivers and providers when a child progresses into a state of higher risk, allowing early intervention (e.g., giving medication or safety proofing the environment) (24).

In terms of scalability and healthcare deployment, the design of the pipeline—based solely on CPU-compatible and open-source components—makes it suitable for integration into edge computing environments, including wearable devices and portable EEG monitors (15, 20). Unlike supervised deep learning models, which require GPU acceleration and retraining for each patient or site. The workflow generalizes across patients without expert calibration, supporting reproducibility (3, 9). This is valuable for real-time monitoring in ambulatory settings, rural clinics, or home care environments, where computing resources may be limited and consistent neurologist access is not guaranteed.

Importantly, this study targets pediatric EEG, a population that presents unique technical challenges due to high inter-subject variability, developmental effects on EEG rhythms, and increased artifact contamination (5). The successful application of preprocessing techniques—such as ICA for artifact removal and Z-score normalization—demonstrates the robustness of the pipeline in managing noisy pediatric data (14). Furthermore, the consistent clustering structure observed after dimensionality reduction and quality validation (21, 22) suggests that the extracted patterns are not only statistically sound but likely reflect real, underlying neurophysiological states with clinical relevance.

By addressing some limitations in prior EEG clustering literature, the proposed methodology offers a reproducible framework that future researchers can benchmark against. At this point, the present work should be regarded as a methodological step in connecting theory with clinical work. As a proof of concept to guide interpretable seizure prediction, additional validation on larger and diverse patient cohorts is needed before clinical application. While this work offers a straightforward, replicable, and computationally-efficient pipeline for unsupervised detection of pre-ictal EEG microstates in pediatric subjects, several caveats should be discussed to guide future work and application. The restricted age range was from 1.5 to 10 years, and diagnosis may have ecological validity implications and may not well represent the entire range of pediatric epilepsy syndromes. Given that all data were acquired at a single institution—Boston Children’s Hospital, with sequential acquisition from a single scanner, there may also be site-specific biases limiting the generalizability. Future work will need to test the proposed pipeline on larger, multi-center datasets with ethnically diverse pediatric populations and a spectrum of clinical presentations to ensure generalization across recording conditions and epilepsy syndromes.

Although this study primarily treated clusters as static categories, a minimal temporal analysis of consecutive 5-s segments indicated that transitions between states were structured rather than random. Certain clusters, such as 1 and 2, were more likely to evolve into cluster 3, whereas others (e.g., cluster 4 to 1) were rarely observed (Figure 6). This pattern supports the construction that seizure onset may involve preferred trajectories through metastable EEG states. While preliminary, these observations strengthen the case for extending the pipeline with formal temporal modeling approaches such as Hidden Markov Models, recurrence plots, or state-space reconstructions in future work. The models would align with dynamical systems perspectives of epilepsy, where seizures are understood as transitions across metastable attractor states, and could improve prediction accuracy by detecting trajectories rather than isolated states.

The study demonstrated that seizures emerge from slow transitions across metastable brain states within a dynamical systems framework (28). Future extensions of this work should incorporate temporal modeling approaches—such as Hidden Markov Models, recurrence plots, or dynamic graph-based methods. To capture the evolving trajectory through microstates, thereby enriching the predictive value of the clustering outputs.

The present investigation was limited to pre-ictal EEG epochs and was not compared to inter-ictal or post-ictal epochs. Although this circumvents possible class imbalance, it restricts the capacity to establish whether the microstates identified are genuinely unique for the pre-ictal state. Incorporating control states in subsequent pipeline steps might give a framework to differentially label EEG states that are both discriminant and time-wise predictive of seizures.

The statically extracted features treat all 5 s independently and do not explicitly model the dynamics within the window. In the future, time-varying features (for example, phase-amplitude coupling, microstate duration, or spectral evolution) can be introduced to capture the nonlinear temporal dynamics of seizure precursors more effectively.

The physiologic designation of each identified cluster is left to human interpretation. It is not possible to give an absolute identification of clusters with functional or clinical labels without the ground truth. Future investigation could conduct the *post hoc* annotation of cluster types using expert input, behavioral correlates to enhance interpretability, and clinical translation.

The proposed pipeline, while computationally inexpensive and CPU compatible, has not been evaluated in online clinical applications. Future studies using the pipeline will be possible with wearable EEG devices of the future or the early version of a bedside monitoring system in evaluating the pipeline’s latency and robustness. Integration with edge computing or mobile health platforms will require adaptation for streaming data and continuous analysis.

Children exhibit dynamic developmental changes in cortical rhythms, especially over months or years. Because the model does not account for developmental changes, future research should explore adaptive frameworks that recalibrate clustering as a child’s EEG matures, ensuring long-term reliability.

These sources of future research are consistent with the recently proposed dynamical systems framework for seizure generation (30). The paper posits that seizures occur through progression in high-dimensional state space via bifurcations and transitions. The unsupervised clustering pipeline provides a platform for empirically detecting these hidden microstates. However, it needs state-space reconstruction, attractor modeling, and bifurcation analysis to formally represent system dynamics leading up to ictal onset. This type of integration would connect theoretical models and empirical EEG data, and may result in more accurate and actionable seizure prediction systems (31).

## 5 Conclusion

This work presented the proposed workflow for unsupervised clustering of pre-ictal EEG data in pediatric epilepsy. Using a structured, five-stage workflow—comprising data segmentation, signal preprocessing, multimodal feature extraction, dimensionality reduction, and K-Means clustering—the work demonstrated the ability to uncover robust and physiologically meaningful microstates that precede seizure onset. Notably, the method operates entirely on CPU-based resources and requires no expert-labeled data, making it highly suitable for real-time deployment in diverse clinical and resource-limited environments.

The approach identified four distinct clusters of pre-ictal EEG segments, which proved stable across multiple validation metrics. The pipeline also addressed many common shortcomings of the EEG clustering literature by not requiring patient-specific tuning, consistently reporting clustering quality. Also, it was applied to a homogeneous pediatric population.

The ictal EEG microstates discovered herein probably correspond to transitional microshifts in cortical excitability and connectivity. They are consistent with the dynamical systems model of ictogenesis. In the clinical context, this model can support next-generation label-free and generalizable seizure forecasting systems that can be integrated into wearables or ambulatory EEG systems. The low computational burden and generalizability of the method is especially appealing for children who often impose compliance and signal quality issues.

One principal interpretation of these clusters is still reasonably speculative. *Post hoc* expert annotation, behavioral correlates (e.g., reported pre-seizure symptoms) or multimodal biomarkers (e.g., fMRI, autonomic signals) could be used to validate the external meaning of each cluster. Shaping into the model’s 3D shape in this way would improve clinical interpretability and guarantee that the detected microstates are related to functionally meaningful brain states.

In conclusion, the study is a methodological step forward under the constraint of clinical utility in EEG-based seizure prediction. It forms the basis for future studies of unsupervised EEG state discovery and paves the way for the design of interpretable, scalable patient-centered neurotechnology in pediatric neurology. In resource-limited settings or for at-home use, these tools would allow us to monitor the pre-ictal EEG state over a prolonged period of time under general out-of-specialist supervision. This type of system allows caregivers to give rescue medication and ensure that the environment is safe. This possibility of cheap real-time integration highlights the importance of this pipeline in the context of pediatric epilepsy care, where safety and quick intervention are critical.

## Data Availability

This study utilized the publicly available, de-identified CHB-MIT Scalp EEG Database, collected by the Children’s Hospital Boston (12)and hosted on PhysioNet (23).
